# Imaging Outcomes of MRI After CT in Pediatric Spinal Trauma: A Single-center Experience

**DOI:** 10.1097/BPO.0000000000002765

**Published:** 2024-07-09

**Authors:** Aapo Sirén, Mikko Nyman, Johanna Syvänen, Kimmo Mattila, Jussi Hirvonen

**Affiliations:** * Department of Radiology, University of Turku and Turku University Hospital, Turku; † Department of Pediatric Orthopedic Surgery, University of Turku and Turku University Hospital, Turku; ‡ Medical Imaging Center, Department of Radiology, Tampere University and Tampere University Hospital, Tampere, Finland

**Keywords:** computed tomography, imaging, magnetic resonance imaging, pediatric, spine, trauma

## Abstract

**Background:**

Imaging has an essential role in the diagnostic workup of suspected pediatric spinal trauma. The most suitable imaging method is still being debated and needs to be considered regarding the patient, injury, and local resources. Magnetic resonance imaging (MRI) is often performed after computed tomography (CT) in case of neurological symptoms or suspected ligamentous disruption. However, it is unclear if the MRI yields significant additional value after CT if the spinal cord injury is not suspected and if the MRI could be used as the sole imaging modality in an emergency department. This study aimed to assess the diagnostic value of emergency MRI after CT in suspected spinal trauma in children and adolescents.

**Methods:**

The imaging data and medical records of patients 17 years of age and younger with emergency spinal CT and MRI over 8 years were retrospectively reviewed. The primary study outcome was the diagnostic accuracy of the 2 imaging modalities in detecting surgically treated spinal injuries.

**Results:**

The study population consisted of 100 patients. Computed tomography and magnetic resonance imaging demonstrated all 7 surgically treated injuries, although one of the injuries was initially missed with CT. Magnetic resonance imaging revealed more injuries, but none of the injuries visible on CT required surgical fixation. Magnetic resonance imaging was able to exclude unstable injuries in patients who had highly suspicious or unequivocal findings on CT.

**Conclusions:**

Magnetic resonance imaging and computed tomography were both highly accurate in detecting unstable pediatric spinal injuries requiring surgical treatment. Magnetic resonance imaging seems not to reveal additional unstable injuries after adequately interpreted spinal CT.

**Level of Evidence:**

Level III—retrospective observational study.

Medical imaging has an essential role in the diagnostic workup of suspected pediatric spinal trauma, even when the best current clinical decision rules are used.^1–4^ The most suitable imaging method is still being debated. Conventional radiographs are widely recommended,^5^ but their diagnostic accuracy has been questioned.^6,7^ The use of computed tomography (CT) has increased^8^ because of its presumed better accuracy^9^ but with the cost of higher radiation dose.^10^ Magnetic resonance imaging (MRI) offers a highly sensitive alternative for detecting spinal injuries^11–14^ without exposing the patient to ionizing radiation.

In our institution, we have widely used emergency MRI in pediatric spinal trauma as a first-line imaging modality. MRI has been shown to exclude injuries requiring surgical treatment—moreover, complementary CT after MRI did not yield consequential additional findings to those already detected on MRI.^14^ In the current study, we aimed to retrospectively examine the complementary clinical value of MRI after CT in detecting unstable spinal injuries in children and adolescents in the emergency department using surgical treatment as a reference standard. We also assessed the overall diagnostic accuracy of CT using MRI as a reference standard and took the experience level of the reporting radiologist into consideration. Our hypothesis was that in the pediatric population, the accuracy of MRI in detecting spinal injuries requiring surgical treatment is at least comparable to that of CT.

## METHODS

We retrospectively reviewed the charts of patients who had undergone an emergency spinal MRI at our institution between April 1, 2013, and August 31, 2021, a time period for which we had a research permit. Our hospital is an academic tertiary care referral center for 470,000 people. The inclusion criteria for the study were (1) spinal MRI due to acute trauma, (2) spinal emergency CT ≤5 days before spinal MRI, and (3) age 17 years or less.

When we compared the accuracy of MRI and CT in detecting surgically treated injuries, surgery was defined as (1) internal fixation or (2) halo bracing. The use of a rigid cervical collar or thoracic extension brace was not included in the analysis because of the need for more evidence regarding their optimal use in injuries with equivocal stability.^15–19^ We also assessed the diagnostic accuracy of spinal emergency CT in detecting any sign of acute trauma using spinal MRI as a reference standard. The impact of the radiologist’s level of expertise on the diagnostic accuracy of CT was evaluated. The analysis did not include the image interpretation of responsible pediatric orthopaedic surgeons because the surgeons’ image reviews were not systematically recorded in the patient charts. To see if there were primarily missed unstable injuries requiring delayed surgery, we searched for the medical records of the patients included in the study. Our hospital is the only center within the district providing pediatric spinal surgery. Hence, in our health care system, assuming that injuries requiring delayed surgical treatment would have emerged in the medical records is justified. The follow-up time was defined as the time between the emergency MRI and the last date the patient was known to reside within our hospital district.

The emergency MRI scans were performed with a Philips Ingenia 3-T system and Philips dStream coils (Philips Healthcare, Best, Netherlands). The standard MRI protocol included sagittal T1-weighted, sagittal and axial T2-weighted, sagittal and coronal short tau inversion recovery (STIR), sagittal diffusion-weighted, and sagittal gradient-echo T2*-weighted. In selected cases, dedicated imaging of the level C0-C2 was performed with a small field of view proton density- and T2-weighted series. Emergency CTs were performed with a GE Revolution scanner (GE Healthcare, Chicago, IL) or a Toshiba Aquilition One scanner (Toshiba Medical Systems, Otawara, Japan). In cervical spine CT, a contrast medium was not used. In most cases, the thoracolumbar spine CT was imaged as a part of a whole-body trauma CT; therefore, the cervical spine CT was imaged before administering an iodine-based contrast agent.

This study is a retrospective chart review, and the analysis is based on original radiology reports. A systematic retrospective image review was not performed. Information about the imaging studies, radiology reports, experience level of the reporting radiologist, and patient treatment were extracted from the radiology information system (RIS), picture archiving and communication system (PACS), and medical records.

The results are expressed as the number of cases (*n*), percentages, means, and standard deviations. Ordinal variables were compared with the χ^2^ test. *P* values <0.05 were considered statistically significant. We performed the statistical analyses with IBM SPSS Statistics for Mac (version 28, IBM Corporation, Armonk, NY).

The hospital district board’s permission for the study was obtained. Institutional review board approval nor written patient consent was not needed for the retrospective study.

## RESULTS

One hundred patients met the inclusion criteria. The population characteristics of our study group are reported in Table 1. The mean age was 12.8 years, the median age being 14 years. The most common injury mechanism was motor vehicle accident (45/100, 45%). The mean delay from CT to MRI was 0.8 days (median: 1 d, range 0 to 5 d). There were 6 surgically treated patients. One of them had 2 noncontiguous unstable injuries, having both cervical and lumbar spine treated with internal fixation. Of the 7 surgically treated injuries, 4 were cervical injuries, 2 were thoracic injuries, and 1 was a lumbar injury. Conservative immobilization was used for 42 patients: Glisson’s traction for 6 patients and a rigid cervical collar or thoracic extension brace for 36 patients. Of all patients, 94/100 (94%) had a follow-up time of at least 3 months (median: 49 mo). No delayed need for surgical treatment occurred, indicating that the initial imaging detected all unstable injuries.

**TABLE 1:** Study Population Characteristics

Number of cases	100
Age: mean (SD), range, median	12.8 (4.2), 1-17, 14
Sex: female/male, n (%)	44/56 (44/56)
Type of injury: n (%)	
Traffic accident	45 (45)
Fall	23 (23)
Contacts sports and gymnastics	14 (14)
Horseback riding	7 (7)
Trampoline	6 (6)
Diving	3 (3)
Hanging	2 (2)
Primarily suspicious spine region: n (%)	
Cervical*	82 (82)
Thoracolumbar	18 (18)
Delay from CT to MRI: mean days (SD), range, median	0.8 (1.1), 0-5, 1
Injuries treated with immobilization, n (%)	
Surgical fixation (including halo brace)	7 (7)
Rigid cervical collar or thoracic extension brace	36 (36)
Glisson’s traction	6 (6)
No immobilization	51 (51)

* The field of view of a cervical spine MRI is routinely extended to the level Th4-5 in our department.

**FIGURE 1:**
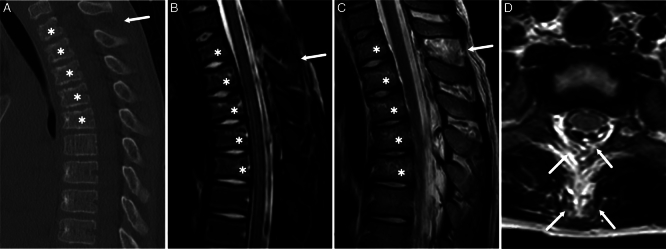
A 12-year-old boy after a trampoline accident. Complete posterior ligamentous complex injury (arrows) in a level Th1/Th2 and compression fractures on vertebrae Th1, Th2, Th3, Th4, and Th5 (asterisks). (A) Noncontrast computed tomography, sagittal plane, (B) Magnetic resonance imaging (MRI), short tau inversion recovery, sagittal plane, (C) MRI, T2-weighted, sagittal plane, and (D) MRI, T1-weighted, axial plane.

Most CTs and MRIs were reported by a board-certified radiologist with a fellowship-trained subspecialization in neuroradiology, musculoskeletal radiology, or emergency radiology with more than 7 years of experience in radiology (Table 2). Magnetic resonance imaging readers accurately reported all 7 injuries requiring surgical treatment. Computed tomography reports were accurate in 6 cases, but in 1 out of 7 (14%) surgically treated injuries, the unstable features of the injury were missed (Fig. 1). In this case, an MRI was performed to further evaluate the wedge-shaped thoracic vertebrae. In addition to edematous compression fractures, MRI revealed a posterior ligament complex (PLC) injury requiring internal fixation. When this case was retrospectively reviewed, the widened interspinous distance could be noted; therefore, the suspicion of an unstable injury should have been raised already on CT.

**TABLE 2:** Training Level of Reporting Radiologist, n (%), and the Proportions of Correct Computed Tomography Interpretation in Different Experience Groups

	In training (>3 y of experience in radiology)	Board-certified radiologist (>5 y of experience)	Fellowship-trained neuro-, musculoskeletal- or emergency radiologist (>7 y of experience)
CT reports	25 (25)	20 (20)	55 (55)
MRI reports	1 (1)	16 (16)	83 (83)
	In training	Board-certified radiologist	Board-certified neuro-, musculoskeletal- or emergency radiologist
True-positive or true-negative findings in CT	14 (14)	12 (12)	40 (40)
False-positive or false-negative findings in CT	11 (11)	8 (8)	15 (15)

The results of comparing the diagnostic accuracy of CT in detecting any findings related to acute trauma using the MRI as a reference standard are presented in Table 3. The CT report accuracy was trending higher among the more experienced readers (Table 2), but this association did not reach statistical significance.

**TABLE 3:** Diagnostic Accuracy of Computer Tomography in Any Finding Related to Acute Trauma Using Magnetic Resonance Imaging as a Reference Standard

True positive	35
True negative	27
False positive	25
False negative	13
Sensitivity	0.73
Specificity	0.51
Positive predictive value	0.57
Negative predictive value	0.68
False positive rate	0.48
False negative rate	0.27
Accuracy	0.61

In 16 patients, MRI revealed bone marrow edema in a vertebra not identified as injured on CT. None of these injuries were unstable. In 7 patients, MRI did not show bone marrow edema in a vertebra that was misinterpreted as compressed on CT. As expected, no soft tissue injuries or nondisplaced ligamentous injuries were reported on CT.

## DISCUSSION

In our study population of 100 pediatric trauma patients, CT and MRI were accurate in detecting unstable spinal injuries. All (7/7) unstable and surgically treated injuries were reported with MRI, while 1 unstable injury was missed on the CT report. However, the unstable features of the missed PLC injury could also retrospectively be seen in CT. Our results of the high detection rate of surgically treated injuries with both modalities align with the previous studies on the subject.^11,13,20–24^ The patient whose unstable upper thoracic spine injury was missed on CT did not have a high-energy trauma (Fig. 1). He was hurt in a trampoline accident and reported midline tenderness at the level of the cervicothoracic junction. The patient was referred to MRI to confirm whether the slightly wedge-shaped thoracic vertebrae were edematous, suggesting acute bony injury, but MRI also demonstrated an unstable PLC injury.

Henry et al^12^ studied the diagnostic accuracy of CT and MRI in pediatric cervical spine trauma using CT as a reference standard for osseous injuries and MRI as a standard for soft tissue injuries. They found excellent sensitivity of MRI for osseous injuries but low sensitivity of CT for soft tissue injuries.^14^ When we assessed the sensitivity and specificity of CT regarding any traumatic findings using MRI as a reference standard, CT’s sensitivity was 0.78 and specificity was 0.51. The false-positive rate was as high as 0.48 (Table 3).

**FIGURE 2:**
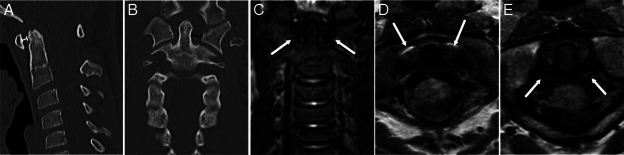
A 10-year-old male fell 2 m head first and reported severe neck pain. The atlantodental interval was reported as slightly widened (3.2 mm, capped white line), and the left-sided odontoid-lateral mass interval was also noted as widened (5.5 mm, capped black line). However, on magnetic resonance imaging (MRI), no edema is seen around the odontoid [image (C), white arrows] and the alar ligaments [image (D), white arrows] and the transverse ligament [image (E), white arrows] are intact. (A) Noncontrast computed tomography (CT), sagittal plane, (B) Noncontrast CT, coronal plane, (c) MRI, short tau inversion recovery, coronal plane, (D) MRI, proton density, axial plane, and (E) MRI, proton density, axial plane.

**FIGURE 3:**
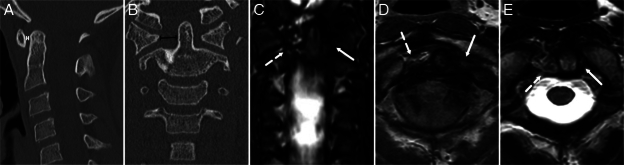
A 10-year-old male was injured in a contact sport and reported slight neck pain in the level C4-C5. The atlantodental interval was reported as normal (1.5 mm, capped white line), but the right-sided odontoid-lateral mass interval was widened (8.9 mm, capped black line). On magnetic resonance imaging (MRI), right-sided edema is seen around the odontoid [image (C), broken white arrow]. The right alar ligament was torn [image (D), broken white arrow], and the transverse ligament was partially ruptured on the right side [image (E), white broken arrow]. Solid white arrows on images (C), (D), and (E) annotate intact structures on the opposite side of the injury. (A) Noncontrast computed tomography (CT), sagittal plane, (B) Noncontrast CT, coronal plane, (C) MRI, short tau inversion recovery, coronal plane, (D) MRI, proton density, axial plane, and (E) MRI, T2-weighted, axial plane.

Our sample’s most common incorrectly suspected injury was an upper cervical spine injury (Fig. 2, Table 4). Considering the potential fatality of these injuries, all patients with a suspected upper cervical spine injury should be referred to further imaging (Fig. 3).^9,25^ MRI can solve most problems in these cases, whereas the high false-positive (and negative) rate of CT leads to a significant amount of complementary imaging with the 2 modalities. In a recent study by Stephenson et al,^24^ retroclival hematoma was a connecting finding in unstable craniocervical junction injuries and was also present in injuries missed in CT reports. Whether retroclival hematoma is frequently found in unstable upper cervical spine injuries is unknown. However, it should be subject to additional studies as a possible improvement to the sensitivity of CT in craniocervical junction injuries.^26^ There were no surgically treated craniocervical junction fractures in our study sample. Still, we had patients with upper cervical spine injuries (occipital condyle avulsion fracture, unilateral type 2 Jefferson fracture, type 2 hangman fracture, alar ligament avulsion, and transversal ligament avulsion) managed with a rigid cervical collar. None of these patients had a retroclival hematoma.

**TABLE 4:** Types of Missed and Erroneously Suspected Injuries on CT Reports

	Fracture or bone contusion	Posterior ligament complex injury	Atlantoaxial joint injury	Soft tissue
False-negative CT	1	5	5	2
False-positive CT	7	3	15	0

According to our results and previously published data, the traumatic findings detected only in MRI but not in CT are unlikely to be unstable. Most stable injuries like bone bruises, nondisplaced fractures, and PLC edema without complete disruption do not need surgical treatment. Should one use expensive, time-consuming, and less available MRI instead of CT in neurologically intact patients if both can reveal unstable injuries? Ideally, we should have more precise clinical decision rules to reduce imaging rates and accurately scan the most suspicious patients with MRI. In reality, MRI is a worthy option for CT for clearing the pediatric spine because of its high sensitivity and specificity achieved without ionizing radiation. It is also the sole imaging modality demonstrating spinal cord injury without radiographic abnormality (SCIWORA).^18,27,28^ MRI is not a silver bullet, but still a versatile tool for diagnostic workup of spinal trauma.^29^ With emergency MRI, it is possible to reduce the follow-up imaging after a pediatric spinal trauma,^30^ and in certain circumstances, emergency MRI is estimated to be cost-effective in clearing the cervical spine of obtunded pediatric patients.^31^ However, a larger-scale cost-effectiveness analysis of the different imaging modalities in pediatric spinal trauma remains to be done.

Medical imaging with ionizing radiation is thought to be associated with increased future cancer risk.^10,32^ However, the association and its biological basis established with high-dose radiation have been questioned, considering the low-dose radiation used in imaging.^33,34^ A recent large-scale multinational study by Bosch de Basea Gomez et al^35^ strived to take account of the deficiencies of the earlier epidemiological studies. They still found an increased risk of hematologic malignancies among the population having undergone CT imaging as a child, but the risk for solid cancer remains unclear. Modern CT technology with iterative- or deep learning–based image reconstruction helps lower radiation doses, but the doses should not be reduced at the expense of image quality.^36^ MRI requires sedation or anesthesia in young and restless children. Anesthesia demands resources and is not without risks. Even if most children aged 5 years or older can undergo MRI without sedation,^14,37,38^ the most appropriate imaging modality always depends on the patient, type of injury, and local resources.^39^ Currently, no comparison exists between the potential long-term adverse effects of ionizing radiation and anesthesia. Our study shows that diagnostic quality is not an issue in clinical reality, but the decision between CT and MRI needs to be made on other grounds.

We found a nonsignificant trend toward higher diagnostic accuracy of CT among fellowship-trained radiologists versus general radiologists or residents. Hassan et al^40^ showed that pediatric cervical spine trauma CT sensitivity was better among subspecialized radiologists. In the era of defensive medicine and growing numbers of CT imaging in trauma centers,^41,42^ inaccurate reporting leads to the rapid growth of complementary imaging with a subsequent human and economic burden.^8^ This underlines the role of education and the need for experienced staff in the emergency departments and outside office hours. Diminishing unnecessary scans would also reduce the number of unequivocal findings and false positive reports, especially those related to pseudosubluxations and other relatively common variants of the normal anatomy.^43,44^

Our study has many limitations, the most obvious being its retrospective nature and the lack of a systematic retrospective image review. We do not know how many minor traumatic changes could have been seen with the CT in a careful re-evaluation and how many false positives could have been avoided using only experienced readers. However, knowing MRIs previously demonstrated high diagnostic accuracy in spinal trauma,^11^ our focus was on assessing the diagnostic yield of CT and MRI in an actual clinical setting, as the standards of retrospective image analysis can seldom be reached in the emergency setting. We could not include the image review of the responsible pediatric orthopaedic surgeon in the analysis of diagnostic accuracy; still, the decisions about the treatment were based on the surgeon’s verdict. A future study with retrospective image analysis involving pediatric orthopaedic surgeons would be highly valuable. The MRI studies included in the analysis were interpreted mostly by fellowship-trained radiology subspecialists, and it is unclear if the diagnostic accuracy would have been as good if the readers had been less experienced. Finally, regarding generalizability, emergency MRI may not be available in all institutions, and diagnostic accuracy may not be adequately high during on-call hours by less experienced radiologists. Despite the limitations, our results reflect the real-life diagnostic yield of MRI after CT in an emergency department of a small tertiary care trauma center.

In conclusion, we found the diagnostic yield of both CT and MRI excellent in excluding unstable spinal injuries in children and adolescents. However, according to our results and previously published data, the traumatic findings detected only in MRI but not in CT are unlikely to be unstable, suggesting the additional value of MRI after unremarkable CT being low. Comprehensive cost-effectiveness and long-term safety analysis regarding radiation-related and anesthesia-related risks are required to assess which one would be the most suitable first-line cross-sectional imaging in pediatric spinal trauma.
